# Large-sample neutron activation analysis in mass balance and nutritional studies

**DOI:** 10.1017/jns.2018.6

**Published:** 2018-04-12

**Authors:** Albert Van de Wiel, Menno Blaauw

**Affiliations:** 1 Delft University of Technology, Faculty of Applied Sciences, Radiation Science and Technology, Clinical Medicine and Isotopes for Health, Mekelweg 15, 2629 JB Delft, the Netherlands; 2 Reactor Institute Delft, Mekelweg 15, 2629 JB Delft, the Netherlands

**Keywords:** Isotopes, Neutron activation analysis, Trace elements, Mass balance studies, Innovative techniques, ICP, inductively coupled plasma, LS, large-sample, NAA, neutron activation analysis

## Abstract

Low concentrations of elements in food can be measured with various techniques, mostly in small samples (mg). These techniques provide only reliable data when the element is distributed homogeneously in the material to be analysed either naturally or after a homogenisation procedure. When this is not the case or homogenisation fails, a technique should be applied that is able to measure in samples up to grams and even kilograms and regardless of the distribution of the element. An adaptation of neutron activation analysis (NAA), called large-sample NAA, has been developed and proven accurate and may be an attractive alternative in food research and mass balance studies. Like standard NAA, large-sample NAA can be used to measure both toxic and trace elements relevant for nutrition.

## Introduction

Deficiencies of essential elements such as Fe, Zn and Cu are a major health problem both in developed and underdeveloped countries. They may be caused by insufficiencies in food quality, -pattern or -intake or by disorders affecting resorption and metabolism. Information on the actual bioavailability of such elements in meals and food products can be obtained by mass balance studies. In such studies it is crucial that the concentration of those elements is measured very accurately both at the site of intake and at the sites of excretion, such as in urine and faeces. Usually small samples, e.g. 1 g or less, are obtained from food and excretion products (faeces and urine) and used for analysis. However, reliable data will only be obtained when the element of interest is distributed homogeneously and the subsample is truly representative. If that is not the case or homogenisation procedures fail, usual techniques for the measurement of low concentrations of elements such as advanced forms of MS cannot be applied. Large-sample neutron activation analysis (LS-NAA) offers an appropriate alternative.

## Mass balance studies

Mass balance studies are used to obtain information on the actual bioavailability of major and trace elements present in meals and food products. Such studies do not usually focus on one meal or one food product but cover a longer period, e.g. 5–7 d in which 8–10 kg of food and 10–14 litres of drinking solutions are consumed. To quantify the intake of a particular element it is common to use the double-portion method: one portion is consumed by the test person while another identical portion is used for the analysis and measurement of the specific element. The intake of an element is calculated either by adding up the amounts of that element present in each component of the food or by homogenising the entire intake and analysing representative subsamples. The quality of the homogenisation can be checked by analysing more (e.g. fifteen) small test portions. However, there are situations where the distribution of an element is not homogeneous or homogenisation procedures are problematic. For instance, it is not unusual to use freeze-drying as part of the homogenisation process. However, if meat is freeze dried, the resulting product is quite a hard piece that cannot be easily crushed in usable small parts. On the other hand, freeze-dried sweet fruits can be easily crushed, but the material, still containing sugar, gets very sticky once liquid N_2_ is poured on it^(^^1^^)^. Most techniques to measure low concentrations of elements, such as inductively coupled plasma MS (ICP-MS), only use small samples. In recent years the Radiation, Science and Technology department of the Delft University of Technology (TU Delft) has worked intensively on the adaptation of the NAA facility, making it possible to measure even low concentrations of elements in large samples up to kilograms.

## Neutron activation analysis

NAA is a technique for qualitative and quantitative multi-element analysis of major, minor, trace and rare elements in all kinds of materials including those from human origin such as blood, nails, hairs and tissue samples. The method is based on the bombardment of a sample with neutrons followed by capture of a neutron by the nucleus of an element and subsequent conversion to a radioactive isotope. NAA requires a source of neutrons, ideally a nuclear reactor.

**Fig. 1.**
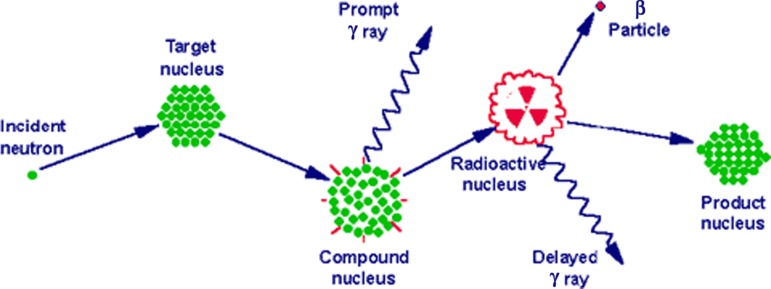
Basic concept of neutron activation analysis.

Samples are first encapsulated in a vial made of either high-purity linear polyethylene or quartz. Samples and standards are then packaged and irradiated in the reactor at a constant, known neutron flux. The research reactor uses uranium fission providing a high neutron flux. The type of neutrons generated are of relatively low kinetic energy (KE), typically less than 0·5 eV, and are termed thermal neutrons. However, experimental parameters can be varied resulting in neutrons with a moderate (0·5 eV–0·5 MeV) or high (>0·5 MeV) KE. Upon irradiation, a thermal neutron interacts with the target nucleus of an isotope of an element via a non-elastic collision, causing neutron capture. This collision forms a compound nucleus which is in an excited state. This state is unfavourable and the compound nucleus will almost immediately de-excite into a more stable configuration through the emission of a prompt particle and one or more characteristic prompt γ photons. In most cases, this more stable configuration yields a radioactive nucleus. The newly formed radioactive nucleus now decays by the emission of both particles and one or more characteristic delayed γ photons (Fig. 1). This decay process is at a much slower rate than the initial de-excitation and is dependent on the unique half-life of the radioactive nucleus. The radioactive emission characteristics and decay paths of the various elements are well known. They are usually measured with a semiconductor detector utilising the semiconducting element germanium. The intensity of the radioactive emissions is proportional to the number of nuclei of the element and based on the information of the spectra of emissions, concentrations of elements, present in the sample, can be calculated. Until the introduction of particle-induced X-ray emission and ICP atomic emission spectroscopy and ICP-MS, NAA was the standard analytical method for performing multi-element analyses with minimum detection limits in the sub-parts per million range^(^^2^^)^. The technique is non-destructive, the chemical structures of the sample stay intact, and there is no need to convert and/or dilute a sample into a suitable solution prior to analysis with inherent risks of contamination and element loss. Apart from this simple preparation, it is a highly accurate technique meeting the requirements of a primary method of measurement^(^^3^^)^. Drawbacks, however, are that the irradiated samples may remain radioactive for many years after the initial analysis, requiring handling and disposal protocols and that the number of research reactor facilities has declined over the years. This may explain why its use in clinical medical research has been limited while much progress has been made in MS techniques, which also have easier access.

## Large-sample neutron activation analysis

**Fig. 2.**
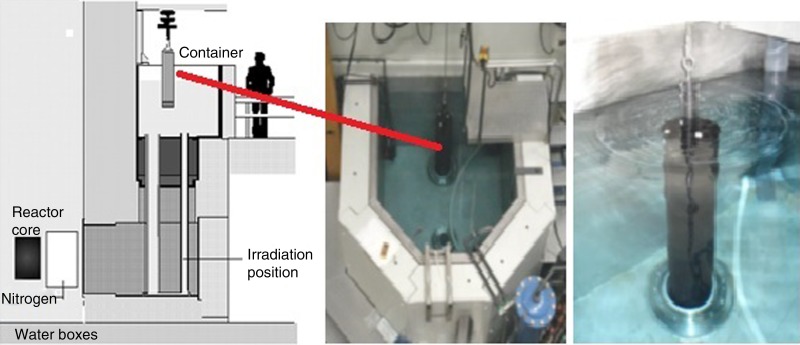
Set up of the large-sample neutron activation analysis facility.

Conventional NAA of human material usually deals with freeze-dried samples of 50–200 mg. In the case of LS-NAA, samples are positioned inside a graphite cylinder, that can contain kilograms of material. The sample is surrounded by eighty neutron flux monitors (Zn foils) positioned in a fixed grid in the walls of the graphite cylinder. The cylinder is then positioned in the thermal column of the reactor (Fig. 2). Irradiation time is usually longer than in small-sample NAA and also the measurement facility is adapted with a greater distance between sample and detector endcap. Although the accuracy and reliability of this method has been proven in the years after its development, the method has not gained much attention in clinical research^(^^4^^,^^5^^)^. Recently, new interest in the technique has been shown by Yagob *et al.*^(^^6^^)^ measuring Fe concentrations in commercially available microwave meals and in porridge fine wheat grain products. Comparing standard small-sample NAA with the LS technique showed equivalent results in the measurement of Fe in the porridge fine wheat grain. Measurement of Fe in merged microwave meals containing all sorts of food was not met with analytical problems such as radiolysis or gas formation during irradiation. In other experiments the same research group has shown that NAA can be applied for Fe measurements in blood, blood cells, faeces and urine^(^^1^^)^. This allows the use of one and the same technique to measure even low concentrations of elements in various materials obtained in one experiment.

## Conclusions

Low concentrations of trace elements in human material can be measured with various techniques and great progress has been made in MS during recent years. However, elements are not always distributed homogeneously neither in human tissue nor in food. Homogenisation may produce representative subsamples, but sometimes such a homogenisation procedure fails to deliver reliable material or there is considerable doubt whether samples are truly representative. In that case a technique, such as LS-NAA, that uses all material and does not depend on sampling is an attractive alternative. Like standard NAA, LS-NAA can be applied to measure most toxic (As, Cd) and trace elements relevant to nutrition like Fe, Zn, Cu, Mg and Mn.
